# Primary tumor-associated expression of CXCR4 predicts formation of local and systemic recurrency in head and neck squamous cell carcinoma

**DOI:** 10.18632/oncotarget.22562

**Published:** 2017-11-20

**Authors:** Andreas Knopf, Leila Bahadori, Kristin Fritsche, Guido Piontek, Cord-Christian Becker, Percy Knolle, Achim Krüger, Henning Bier, Yin Li

**Affiliations:** ^1^ Otorhinolaryngology, Head and Neck Surgery, Institute of Molecular Immunology & Experimental Oncology, 81675 München, Germany; ^2^ Institute of Molecular Immunology & Experimental Oncology, 81675 München, Germany

**Keywords:** CXCR4, CXCL12, head and neck squamous cell carcinoma, recurrency, metastasis

## Abstract

**Objectives:**

Despite modern treatment regimens, overall survival in head and neck squamous cell carcinomas (HNSCC) is less than 50% due to local and systemic disease recurrency. The current study aims to identify molecular markers in primary tumor specimens that predict the risk for local and systemic recurrency at the time of initial diagnosis.

**Methods:**

The study included clinic-pathological data of 1,057 HNSCC. MMP2/9, TIMP1/2, CXCR4, and CXCL12 immunohistochemistry was done in 150 randomly selected specimens. For statistics, we employed Chi square, Fisher exact, and Student's t-test. Overall survival (OS) was calculated by Kaplan–Meier and log-rank test. Prognostic variables were subsequently evaluated by Cox regression for forward selection.

**Results:**

CXCR4 positive specimens demonstrated a significant increased risk for tumor recurrency associated death (rT: HR 10.07; p=0.001 / rN: HR 5.04; p=0.013 / rM: HR 2.49; p=0.029) when compared with their unaltered counterparts. Expression of MMP9, TIMP2, CXCR4, and CXCL12 was significantly increased in distant metastasized patients (p<0.0001) and showed significant cross-correlation. In addition, CXCR4 positivity was associated with an increased risk to die due to enhanced T or N status (T1/2 vs. T3/4: HR 5.78; p=0.017; N0 vs. N+: HR 5.18; p=0.033).

**Conclusion:**

CXCR4 positivity in tumor samples at initial diagnosis were associated with reduced overall survival, in particular with respect to increasing T/N status, local and systemic recurrency.

## INTRODUCTION

Head and neck squamous cell carcinoma (HNSCC) is the sixth most common cancer worldwide accounting for approximately 500,000 newly diagnosed cases and 300,000 deaths every year [1, 2]. Tobacco abuse and alcohol consumption, as well as infection with human papillomavirus (HPV), were shown to be the most important risk factors in HNSCC carcinogenesis [3, 4]. Beside HPV status, patient's prognosis is inherently associated with the T, N, M status [5, 6]. Tumor stage-adjusted pre-therapeutic staging determines resectability at primary tumor site and cervical lymph node basin and excludes distant metastasis [7]. Local lymph node metastasis occurs in approximately 60% of HNSCC cases, while distant metastases can be diagnosed in 5% at initial diagnosis [6, 8]. In the absence of distant metastasis, surgery with/without adjuvant (chemo-) radiation and primary chemo-radiation represent the therapeutic mainstay of curative approaches [5, 9]. Curative treatment has to manage the balancing act between sufficient radicality and preservation of functional structures [10–12]. Both, surgical and conservative approaches have been associated with severe comorbidity [10–13]. Despite significant advances in cancer treatment, the 5-year survival rate is less than 50% due to both local relapse and development of distant metastases [4]. While a substantial proportion of HNSCC patients suffer from relapse at primary tumor site and/or cervical lymph nodes, further 15-20% of all HNSCC develop metachronous distant metastases after primary curative treatment, and therefore, become palliative [5, 9]. Thus, it is important to aim for identification of factors allowing prediction of disease recurrence already at the time of initial diagnosis [14]. We recently demonstrated in colorectal cancer that CXCL12 seems to be a decisive factor in the establishment of a pre-metastatic niche in the liver and interference with the CXCL12-CXCR4 axis may inhibit the pre-deposition of a distant organ to attract metastases [15]. In HNSCC, CXCR4 positivity resulted in reduced overall survival (OS) in CXCR4 positive specimens [16]. Molecular mechanisms underlying the worse survival in HNSCC, particularly with respect to tumor recurrency, remain unclear. This prompted us to investigate the impact of proteins associated with the CXCR4-CXCL12 axis (MMP2, MMP9, TIMP1, TIMP2, CXCR4, and CXCL12) in local recurrency and distant metastatic spread of HNSCC. Increased CXCR4 levels correlated with the development of local recurrent disease and distant metastases in HNSCC resulting in a dramatically decreased overall survival.

## RESULTS

### Patient and tumor characteristics

The investigated cohort of 1,057 consecutively surgically and conservative treated HNSCC patients included 841 men and 216 women with a median and mean age of 60 years. The majority of tumors originated in the oropharynx, followed by hypopharynx, larynx, and the oral cavity. While 60% of patients demonstrated lymph node metastasis at the time of diagnosis, M1 status was present in 4%, only. Almost 66% of our patients underwent head and neck surgery with/without adjuvant chemo-radiation. Ninety percent of tumors were assumed to be resected *in sano*, comprising R0 resection in 87% and piecemeal resection (Rx) in 7% of patients. Thirty-four percent of our patients were treated by primary radio(chemo-)therapy (Table 1).

**Table 1:** Epidemiologic and tumor characteristics demonstrate a typical HNSCC cohort with pronounced tumor occurrence in male at 6^th^ decade of life

	HNSCC
**N**	1057
**Age (years)**	
Median [25%; 75%]	60 [53; 67]
Mean ± SD	60±11
**Sex, n (%)**	
Male	841 (80)
Female	216 (20)
**Location**	
Sinunasal	35 (3)
Nasopharynx	20 (2)
Oropharynx	415 (39)
Hypopharynx	220 (21)
Larynx	208 (20)
Oral cavity	154 (15)
CUP	5
**T status, n (%)**	
Tx	8
T1	276 (26)
T2	313 (30)
T3	221 (21)
T4	239 (23)
**N status, n (%)**	
N0	417 (40)
N+	640 (60)
**M status, n (%)**	
M0	1015 (96)
M1	42 (4)
**Grading, n (%)**	
G1	45 (4)
G2	506 (48)
G3	469 (45)
G4	13 (1)
Gx	24 (2)
**R status, n (%)**	
R0	590 (83)
R1	58 (8)
R2	14 (1)
Rx	50 (7)
**Treatment, n (%)**	
OP only	187 (19)
OP + RTX	300 (28)
OP + RCTX	203 (19)
Prim. RCTX	329 (31)
Prim. RTX	36 (3)

Tumors were frequently localized in the oropharynx, hypopharynx, larynx, and oral cavity. While 60% of patients showed lymph node metastasis at the time of diagnosis, distant metastasis was diagnosed in 4%. OP=surgical resection, RTX=radiotherapy, RCTX=radio-chemotherapy.

### CXCR4 positivity is associated with local and systemic disease recurrence

**Figure 1:**
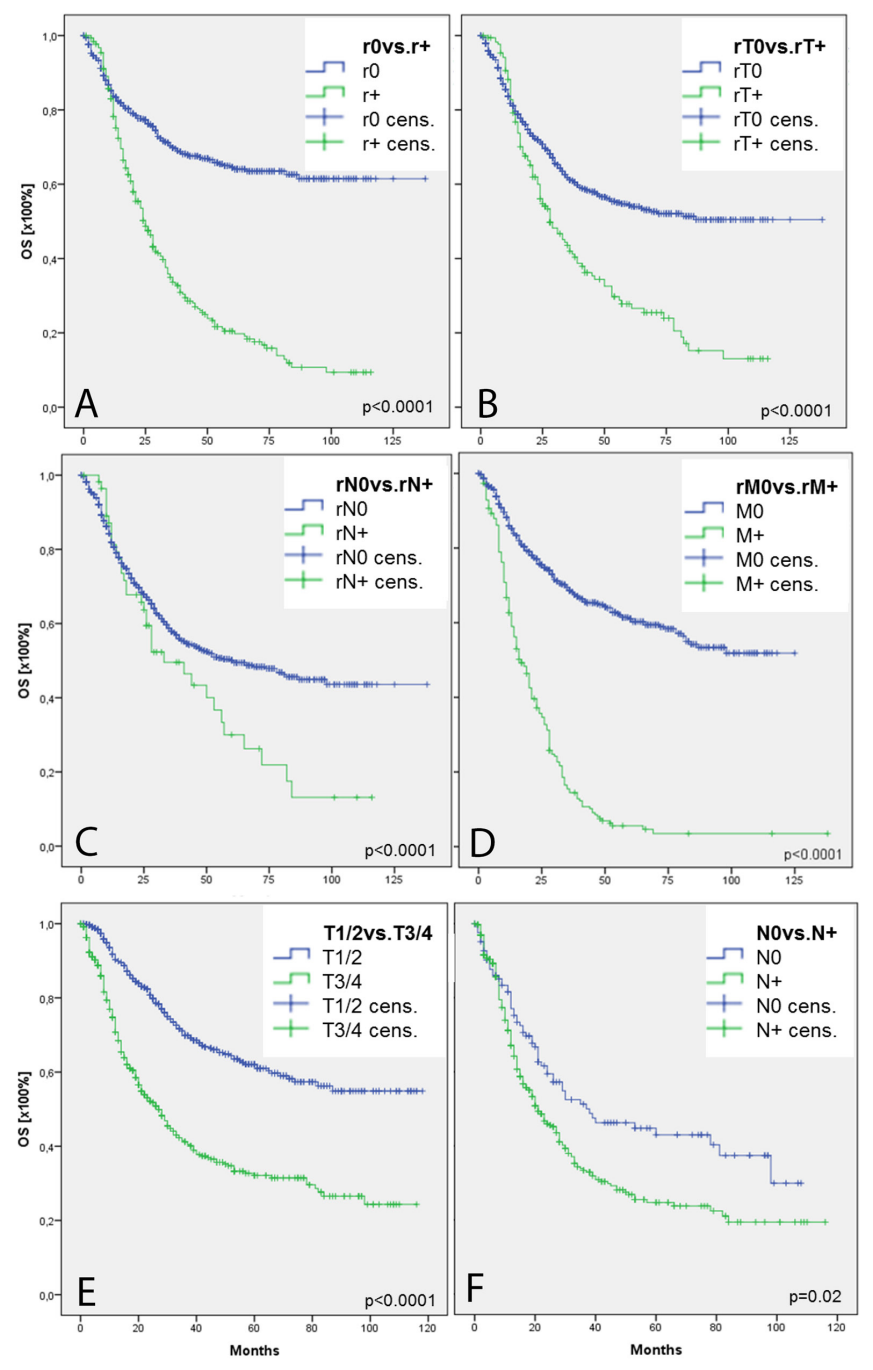
Overall survival in HNSCC Kaplan Maier illustrates overall survival in 1,057 HNSCC patients with respect to tumor recurrency **(A)**. Subgroup analysis demonstrates survival curve in patients with/without recurrency at primary tumor site **(B)**, regional lymph nodes **(C)**, and distant metastasis **(D)**. Fugures **(E and F)** show survival curves in patients with limited (T1/2 or N0) and aggravated (T3/4 or N+) disease burden.

Patients who develop local and/or systemic recurrency demonstrated a significant reduced OS (Figure 1A). While patients without recurrent disease showed a mean OS of 94 months, survival in individuals suffering from recurrency was 38 months (Figure 1A). Subgroup analysis of recurrent disease localization demonstrated a mean OS in patients with rT0 status of 92 months and decreased by recurrency at primary tumor site (rT+) to 44 months (p<0.0001; Figure 1B). OS in patients with recurrency in local lymph nodes (rN+) was 47 months, while mean OS was 76 months in unaltered counterparts (p<0.0001; Figure 1C). Patients with systemic tumor recurrency (rM+) showed the lowest overall survival with 25 months, while patients without systemic disease showed a mean overall survival of 88 months (p<0.0001; Figure 1D). Subgroup analysis of different combination of tumor recurrency (rT+rN0rM0 vs. rT0rN+rM0 vs. rT0rN0rM+ vs. rT+rN+rM0 vs. rT+rN0rM+ vs. rT+rN+rM+) did not reveal differences between the groups (p=0.57; data not shown). Forward selected, proportional Cox regression of survival modifying parameters (T, N, R, G, tumor localization, MMP2/9, TIMP1/2, CXCR4, and CXCL12) identified CXCR4 being the only survival-modifying parameter in HNSCC (Table 2). CXCR4 positivity at primary tumor site was associated with a significantly increased risk for recurrency associated death ranging from HR = 2.49 in patients with systemic disease recurrency, HR = 5.04 in patients with local lymph node recurrency to HR: 10.07 in patients with recurrency at primary tumor site (Table 2). In addition, MMP9-positive patients showed an increased risk for rN+-associated death (HR: 2.25; Table 2). Interestingly, multivariate analysis failed to identify N+ and increasing T status, which traditionally were held to be responsible for local and systemic recurrency-associated death, as independent risk factors. Furthermore, forward-selected proportional cox regression analyzing reduced survival in advanced (T1/2 vs. T3/4) or lymph node positive HNSCC (N0 vs. N+) also demonstrated CXCR4 as solitary risk factor (Table 2). Proportional Cox regression model estimated HR = 5.78 and HR = 5.18 deaths due to increased T status (T1/2 vs. T3/4; p=0.017; Table 2) and lymph node positivity (N0 vs. N+; p=0.033; Table 2). Therefore, patients with advanced T status (T3/4) and lymph node positivity demonstrated significant worse survival when compared with T1/2 (p<0.0001; Figure 1E) and N0 (p=0.02; Figure 1F) status.

**Table 2:** Cox regression of recurrency and T/N status modifying parameters

	HR	95%-CI	p-value
**rT0 vs. rT+**			
CXCR4 pos.	10.07	9.78-11.02	0.001
**rN0 vs. rN+**			
CXCR4 pos.	5.04	4.48-5.24	0.013
MMP9 pos.	2.25	2.02-2.51	0.048
**rM0 vs. rM+**			
CXCR4 pos.	2.49	1.10-5.55	0.029
**T1/2 vs. T3/4**			
CXCR4 pos.	5.78	2.44-6.01	0.017
**N0 vs. N+**			
CXCR4 pos.	5.18	2.40-6.00	0.033

Forward selected proportional Cox regression of 150 randomly selected patients corresponded to survival curves of the entire cohort (Figure 1A-1F). CXCR4 was the only disease modifying parameter. While increasing T and N status failed to be a prognostic parameter in tumor recurrency, subgroup analysis of different T and N status also identified CXCR4 being disease modifying.

### CXCR4 positivity is associated with the development of distant metastasis

After pre-therapeutic staging, 96% of our patients were held to be distant metastasis free, and therefore, underwent curative treatment (Table 1). After a mean follow-up time of 18 months, 14% of these patients were diagnosed with metachronous distant metastasis. The vast majority of patients (82%) showed single organ manifestation, the lungs being the preferential organ of distant metastatic outgrowth (Table 3).

**Table 3:** Distant metastatic profile

	HNSCC
**M stage, n (%)**	
M1 synchronous	42 (4)
M1 metachronous	143 (14)
**Time frame, month (mean ± SD)**	
Diagnosis to metachronous M1	18±14
**Location, n (%)**	
Multi-organ disease	34 (18)
Liver	34 (18)
Lung	88 (48)
Mediastinum	12 (7)
Bone	32 (17)
Skin	16 (9)
Central nervous system	7 (4)
Peripheral lymph nodes	12 (7)
Other	24 (13)

Distant metastasis was diagnosed in 4% at initial diagnosis. After a mean time of 18 months, metachronous distant metastasis occurred in 14%. Lungs were predominantly affected.

**Figure 2:**
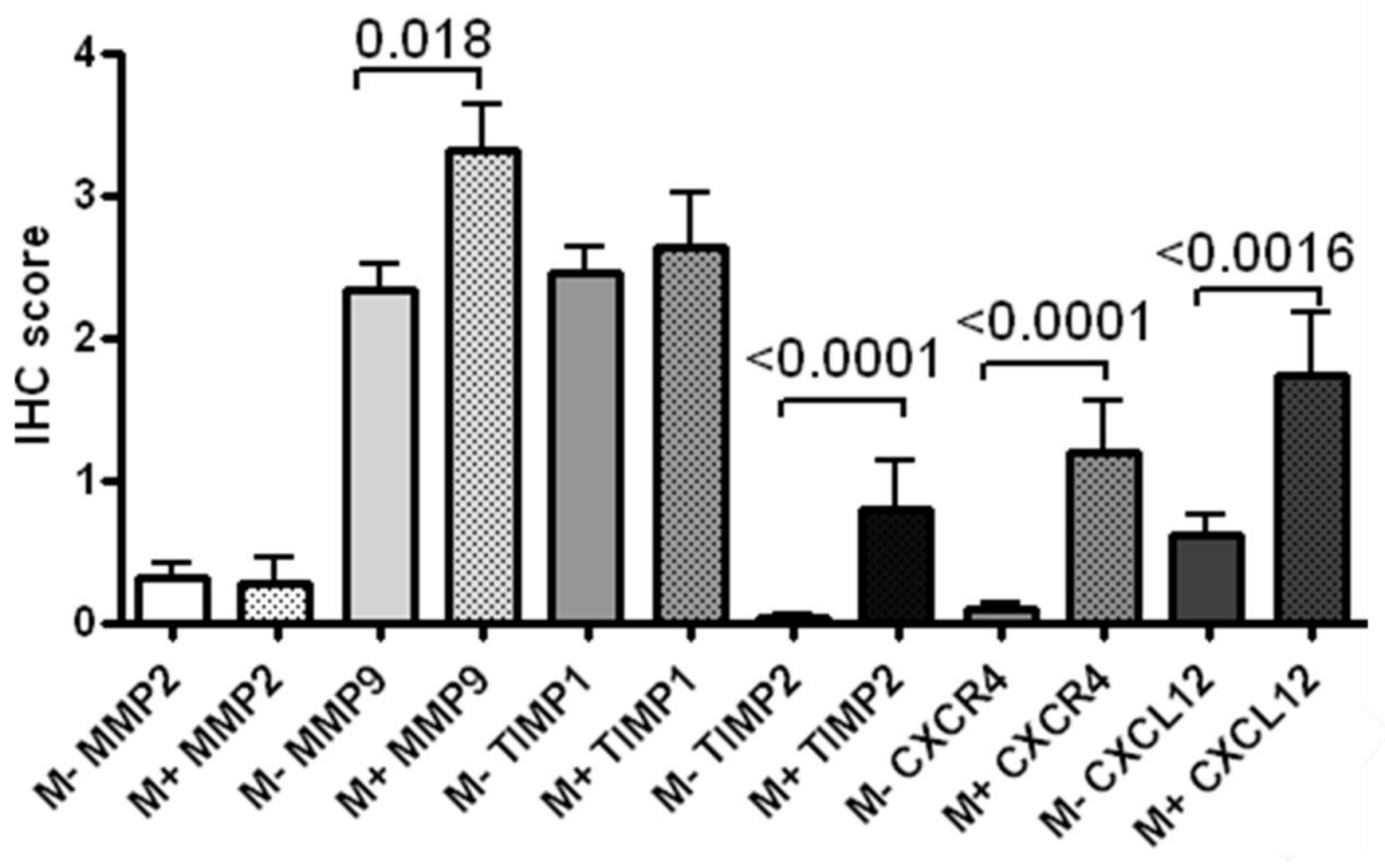
Immunohistochemical expression of markers associated with the CXCR4 axis MMP9, TIMP2, CXCR4, and CXCL12 were significantly higher expressed in distant metastasized patients when compared with their unaltered counterparts.

Immunohistochemistry of CXCR4-CXCL12 axis-associated proteins revealed significantly higher expression of MMP9, TIMP2, CXCR4, and CXCL12 in distant metastasized specimens as compared to their unaltered counterparts (Figure 2). Although FFPE samples were randomly selected from a huge cohort of consecutively included patients with different tumor localization and TNM status, statistical analysis still revealed significant correlation between CXCR4, MMP2 (r=0.2; p=0.04), TIMP1 (r=0.32; p=0.001), TIMP2 (r=0.66; p<0.0001), and CXCL12 (r=0.53; p<0.0001) (Table 4).

**Table 4:** Correlation of immunohistochemical markers

		MMP2	MMP9	TIMP1	TIMP2	CXCR4	CXCL12
**MMP2**	r-value	1	0.003	0.12	0.11	**0.2**	**0.23**
	p-value		0.98	0.2	0.28	**0.04**	**<0.02**
**MMP9**	r-value	0.003	1	0.004	**0.27**	0.12	0.12
	p-value	0.98		0.97	**0.004**	0.23	0.2
**TIMP1**	r-value	0.12	0.004	1	**0.24**	**0.32**	**0.31**
	p-value	0.2	0.97		**0.01**	**0.001**	**0.001**
**TIMP2**	r-value	0.11	**0.27**	**0.24**	1	**0.66**	**0.52**
	p-value	0.28	**0.004**	**0.01**		**<0.0001**	**<0.0001**
**CXCR4**	r-value	**0.2**	0.12	**0.32**	**0.66**	1	**0.53**
	p-value	**0.04**	0.23	**0.001**	**<0.0001**		**<0.0001**
**CXCL12**	r-value	**0.23**	0.12	**0.32**	**0.52**	**0.53**	1
	p-value	**<0.02**	0.2	**0.001**	**<0.0001**	**<0.0001**	

150 randomly selected samples of consecutively included patients showed significant correlation between CXCR4, MMP9, TIMP1, TIMP2, and CXCL12 suggesting functional significance.

## DISCUSSION

Head and neck surgery and primary radio(chemo-)therapy represent therapeutic state-of-the-art in curative HNSCC treatment [5, 7]. Both therapeutic regimens aim to achieve sufficient radicality with a minimum of functional loss. However, all curative approaches are associated with severe patient's morbidity [10, 13]. The current cohort includes a typical HNSCC profile concerning age/gender distribution, primary tumor localization, and TNM status. Therefore, the vast majority of patients (96%) underwent curative treatment. Despite significant advances in HNSCC treatment, the 5-year survival rate is less than 50% due to both local relapse and the development of distant metastases [4]. Tumor stage adjusted pre-therapeutic imaging validates operability at primary tumor site and local lymph nodes and excludes distant metastatic spread. Pre-therapeutic thoracic computed tomography, which was applied in the majority of our patients due to advanced disease status, showed distant metastatic outgrowth in only 4% of patients at the time of diagnosis. After a mean time of 18 months, another 14% of our patients developed metachronous distant metastatic outgrowth, in particular pulmonary metastases. The diagnostic gap in the pre-therapeutic identification of pulmonary metastases was recently demonstrated [17] and highlights the necessity to identify molecular markers that predict both, local and systemic tumor recurrency at the time of initial diagnosis. Recently, CXCR4 came into focus in different tumor entities and CXCR4-positivity was usually associated with local tumor growth, the occurrence of local and systemic metastases and, therefore, reduced survival parameters [14]. However, little is known about the impact of CXCR4-CXCL12 axis in HNSCC. This prompted us to investigate molecular aspects in cohort of 1,057 HNSCC patients with respect to local and systemic tumor disease recurrency as most important indicator in therapy failure. In agreement with Zhao et al. the current study identified CXCR4 as an important survival-associated parameter in HNSCC [16]. CXCR4 positivity of the primary tumor at the time of diagnosis was associated with significantly increased risk for local and systemic recurrency-associated death. Interestingly, advanced T- and N-status did not directly correlate in reduced OS due to development of distant metastasis, but rather due to local tumor progression (T1/2 vs. T3/4). In addition, development of regional metastases (N0 vs. N+) was associated with the CXCR4 status. These results are in agreement with Ishikawa et al. who demonstrate a correlation between CXCR4 expression and development of lymph node metastasis in 90 HNSCC patients [18]. Above that, we could now demonstrate that CXCL12 positivity at primary tumor site was associated with a significant increased risk of lymph node recurrency associated death. In oral cancer, a significantly higher CXCL12 expression was observed in lymph node metastases when compared with the corresponding primary tumor specimens, raising the hypothesis that CXCR4 might be attracted in a paracrine manner on the basis of the CXCL12 gradient from primary tumor towards lymph nodes [19]. More recently, functional aspects of the CXCR4-CXCL12 axis were demonstrated in the establishment of a pre-metastatic niche in the liver [15]. In the current study, protein expression of MMP9, CXCR4, CXCL12, and TIMP2 was significantly higher in distant metastasized individuals when compared with their unaltered counterparts. Although analyzed specimens were randomly selected from a cohort of 1,057 consecutively treated patients, immunohistochemistry revealed correlation between CXCR4, CXCL12, MMP9, TIMP1, and TIMP2. These results agree with the current literature that demonstrated a CXCL12-dependent increase of MMP2 and MMP9 secretion *via* activation of the ERK-1/2 signaling pathway [20, 21]. The association of these markers with the occurrence of distant metastases has already been shown in other tumor entities [19, 22–24]. However, molecular mechanisms of local and systemic disease recurrency in HNSCC mediated by the CXCR4-CXCL12 axis are still delusive at this point.

## MATERIALS AND METHODS

### Patient selection

The current study includes a total of 1,057 HNSCC patients who were consecutively diagnosed in the ENT department of the University Hospital Rechts der Isar, Munich. Tumor samples were histologically reviewed by at least two experienced pathologists. Dysplasia, carcinoma in situ, and other histologic subtypes were excluded. Clinical parameters and survival data were retrospectively collected: age, sex, TNM status (7^th^ edition), grading, treatment modalities, recurrence, and death/loss to follow-up. Patients with lacking data, incomplete staging, and refused/not finished surgical and/or conservative treatment were excluded from survival analysis. The mean follow up time was ≥60 months for all analyzed tumor entities.

### Immunohistochemistry

HNSCC tumor samples were obtained from primary tumor sites at the time of diagnosis. Paraffin-embedded tumor (FFPE) samples from 150 HNSCC were randomly selected from the overall cohort and analyzed via immunohistochemistry (IHC). Subgroup analysis excluded study population driven bias (p = 0.16 – 0.93). FFPE tumor sections (2.5μm) were MMP2 (DCS Innovative Diagnostik-Systeme, Hamburg, Germany, 1:100), MMP9 (Biomol GmbH, Hamburg, Germany, 1:1000), TIMP1 (R&D, Wiesbaden, Germany, 1:500), TIMP2 (Biomol, 1:500), CXCR4 (R&D, 1:200), and CXCL12 (R&D, 1:1000) immuno-stained and visualized with the Bond Polymer Refine Detection Kit (Leica, Nussloch, Germany). Cytoplasmatic expression levels were classified using a scoring system analyzing the staining intensity (0=no staining, 1=low, 2=moderate, 3=strong staining intensity) and the relative proportion of stained tumor cells (0, 1=<10%, 2=10-39%, 3=40-69%, 4=>70 of the tumor cells). A cumulative score (range 0-7 points) was assessed by adding both scores. A positive staining was defined by a cumulative score equal or greater than 3.

### Statistical analysis

Differences between the groups were analyzed using the Chi square test and Fisher exact test for categorical, and the unpaired student's t-test for continuous variables. Correlation between different markers were calculated and expressed by Pearson's r. As main endpoint the overall survival (OS) was assessed measuring the time from treatment to death of any cause. Survival rates and curves were calculated and illustrated by the Kaplan–Meier method and further analyzed by the log-rank test. Variables that revealed prognostic or effect modifying potential on the outcome were subsequently evaluated by the proportional Cox regression for forward selection. p-values <0.05 were considered statistically significant. Statistical analysis was done using SPSS (SPSS Inc., Chicago, IL).

## CONCLUSIONS

CXCR4 positivity in HNSCC is associated with increased risk of local and systemic recurrency associated death. The increased risk can be identified by CXCR4 over-expression at primary tumor site, providing a diagnostic approach to improve treatment stratification.
